# HPV16 E6 and E7 Genetic Variability in Oral and Anal Samples from HIV-Positive MSM

**DOI:** 10.3390/pathogens14121210

**Published:** 2025-11-28

**Authors:** Verdiana Zulian, Silvia Pauciullo, Roberta Sciamanna, Antonio Coppola, Martina De Sanctis, Arianna Borsellino, Paola Del Porto, Franca Del Nonno, Daniele Colombo, Alessandra Scarabello, Anna Rosa Garbuglia

**Affiliations:** 1 Laboratory of Virology, National Institute for Infectious Diseases “Lazzaro Spallanzani” (IRCCS), 00149 Rome, Italy; silvia.pauciullo@inmi.it (S.P.); roberta.sciamanna@inmi.it (R.S.); antonio.coppola@inmi.it (A.C.); martina.desanctis@inmi.it (M.D.S.); annarosa.garbuglia@inmi.it (A.R.G.); 2 Department of Biology and Biotechnology “Charles Darwin”, Sapienza University of Rome, 00100 Rome, Italy; borsellino.1938600@studenti.uniroma1.it (A.B.); paola.delporto@uniroma1.it (P.D.P.); 3 Pathology Unit, National Institute for Infectious Diseases “Lazzaro Spallanzani” (IRCCS), 00149 Rome, Italy; franca.delnonno@inmi.it (F.D.N.); danele.colombo@inmi.it (D.C.); 4 Clinical and Research Infectious Diseases Department, National Institute for Infectious Diseases “Lazzaro Spallanzani” (IRCCS), 00149 Rome, Italy; a.scarabello@inmi.it

**Keywords:** human papillomavirus, HPV16, variant, E6 protein, E7 protein

## Abstract

Persistent infection with high-risk human papillomavirus (HPV), particularly genotype 16, is the main driver of anogenital and oropharyngeal cancers. However, data on HPV16 genetic variability in anal and oral samples from people living with HIV are limited. In this study, we investigated the diversity of HPV16 E6 and E7 genes in anal and oral samples collected from HIV-positive men who have sex with men (MSM) according to different cytological outcomes and anatomical site. Among 53 MSM patients, we obtained 51 E6 and 52 E7 sequences. Lineage A predominated (96.1%), mainly represented by sublineages A1 (84.3%) and A2 (9.8%). The E6 region showed higher variability than E7: 31.4% were identical to the reference (K02718), 51.0% carried the L83V (T350G) mutation, while Q14H, D25N, and D25E were also observed. In E7, only one non-synonymous substitution was detected (N29S). No site-specific variants were detected between anal and oral samples. Overall, these findings confirm the predominance of European A1/A2 variants in Italian MSM and suggest that E6/E7 polymorphisms are not directly linked to lesion grade or anatomical localization.

## 1. Introduction

Cervical cancer is mainly caused by the human papillomavirus (HPV), specifically high-risk (HR) HPV types, including 16, 18, 31, 33, 35, 39, 45, 51, 52, 56, 57, 58, 59, 66, and 68 [1]. The 2022 Global Cancer Observatory of the World Health Organization’s (WHO) report indicated that cervical cancer is the eighth most diagnosed cancer globally [2,3]. HPV 16 alone is estimated to cause 2/3 of all cervical cancer cases [4,5]; it is also considered the main cause of anal and oropharyngeal cancers. Epidemiologic studies show that the incidence of anal cancer increased by 2.7% from 2001 to 2015 and mortality also increased 3.1% [6]. The risk for people living with HIV (PLWH) is over 100 times higher than the general population [7] and the relative risk to develop anal cancer is 1.5–2-fold higher for PLWH than in the general population [8]. Among PLWH, men having sex with men (MSM) are still at a higher risk than PLWH overall, with a 37-fold greater anal cancer risk than the general population [9]. Furthermore, there has been a marked increase in HPV-related oropharyngeal cancer (OPC) over the past 20 years. In the United States, HPV accounts for almost 75% of OPC, and nearly 90% of these tumors have detectable HPV DNA [10,11].

Overall, HPV infection is highly diffused: about 40% of men and women aged 15–59 years in the U.S. are infected with HPV, and high-risk (HR) HPV types are found in 24.2% of men and 19.9% of women [12]. However, about two-thirds of infections resolve within two years without any intervention [13]. The mechanism by which HPV establishes a persistent infection and can cause neoplastic transformation is not fully known/clarified. Despite this, persistent infection with HR HPV types is the primary risk factor for development of high-grade squamous intraepithelial lesions and progression to invasive cancer [14]. HPV16 is the most commonly detected HR genotype in cervical, anal, and oropharyngeal cancers. Some molecular epidemiological studies have indicated that variants of HPV16 may differ in their ability to persist and drive malignant transformation [15]. HPV16 variants were initially classified into five phylogenetic groups—European (E), Asian-American (AA), Asian (As), African-1 (Af-1), and African-2 (Af-2) [16]. More recent classifications define lineages and sublineages on the basis of nucleotide divergence and identify four primary lineages (A–D) and sixteen sublineages (A1–4, B1–4, C1–4, D1–4) [17]. HPV16 lineages show a different distribution across geographical areas. Sublineages A1–A3 are typically referred to as European, A4 as Asian, B1–B4 as African-1, C1–C4 as African-2, D1 as North American, and D2–D3 as Asian-American, while D4 represents a distinct variant within lineage D [18,19]. These sublineages also differ in oncogenic potential. Some of them, such as A4, C, D2, and D3, are more closely associated with cervical intraepithelial neoplasia (CIN) and cervical cancer than others [20]. At the molecular level, E6 and E7 HPV16 oncoproteins are essential to the transformation of cells. Both oncoproteins were consistently expressed in HPV-induced cancers by altering cell cycle regulation. High-risk E6 oncoproteins promote the degradation of the oncosuppressor, TP53, and the pro-apoptotic protein BAK, thereby blocking apoptosis (the process of programmed cell death) and promoting accumulation of mutations within cells, while also inducing telomerase through Myc interactions [21,22]. E7 oncoprotein binds the retinoblastoma protein (pRB) family, promoting the dissociation of E2F transcription factors and cell cycle progression. E6 mutations, specifically E350G or Q14H/H78Y (potentially more prevalent in Asian-American variants), have been associated with higher oncogenic potential and progression from HSIL to cancer [14,23,24,25].

To this point, the majority of studies that focused on the genetic variability of E6 and E7 proteins amongst HPV16 variants have evaluated samples from women with cervical cancer or HSIL. There remains a limited understanding of the genetic variability of HPV16 E6 and E7 in anal lesions, or cases of persistent and transient oropharyngeal HPV16 infections in people, especially those with HIV and among communities at elevated risk for HPV infection.

In this study, we analyzed the genetic variability of HPV16 E6 and E7 in MSM who are HIV-positive and presented low-grade (LSIL) and high-grade (HSIL) lesions or who are negative for intraepithelial lesion and malignancy (NILM). We also evaluated persistent infection of HPV16 in patients with persistent oropharyngeal infections.

## 2. Materials and Methods

### 2.1. Sample and Data Collection

Anal and oral samples were collected from 53 MSM, aged between 29 and 72 years, who attended the National Institute of Infectious Diseases (INMI) “Lazzaro Spallanzani” Hospital in Rome, Italy. Recruitment started in November 2024, and participants were followed up every three months. This study was approved by the local Ethics Committee (ethical approval number 75/2024), and all participants provided written informed consent. All experiments were performed in accordance with the Declaration of Helsinki. During each visit, both anal swabs and oral rinse samples were obtained. The cohort included HIV-positive MSM and who were previously infected by HPV or acquired HPV during follow-up visit. All patients were under antiretroviral therapy (ART).

**Figure 1:**
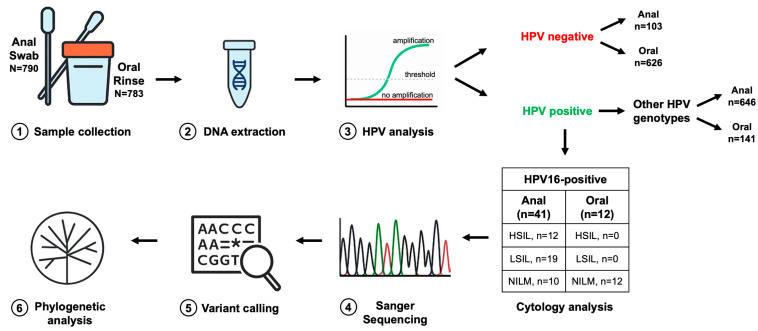
Workflow of the E6 and E7 region analysis. Anal swabs or oral rinse specimens were collected from patients (**1**) and processed for DNA extraction (**2**). Extracted DNA was used for HPV detection and genotyping (**3**) to determine the presence and specific type of infection. Samples identified as HPV16-positive were further characterized through targeted sequencing of the E6 and E7 oncogenes (**4**), in parallel with cytological assessment. Resulting sequences were aligned to the HPV16 reference genome to identify nucleotide variants relative to the consensus (**5**). Finally, phylogenetic analyses (**6**) were performed to reconstruct evolutionary relationships among HPV16 variants and to contextualize sequence diversity within the study cohort. N = number of samples.

In the E6/E7 genetic variability analysis, patients HPV16-positive in anal or oral rinse samples were included, regardless of the cytological findings. These findings ranged from HSIL or LSIL to normal cytology with NILM. In total, 41 HPV16-positive anal samples and 12 HPV16-positive oral samples were selected from 790 anal swabs and 783 oral rinses, respectively (see the methodological workflow in Figure 1). For oral samples, persistence was defined as two consecutive HPV DNA-positive results, or two positive tests within a 24-month period [26]. Thus, all patients harbored HPV16; multiple HPV infections were identified in 38/41 (92.7%) anal samples and 9/12 (75.0%) oral rinses. Among anal samples, 12/41 (29.3%) showed HSIL, 19/41 (46.3%) LSIL, and 10/41 (24.4%) NILM, while all oral samples (12/12; 100%) had NILM cytology, all with persistent HPV infection. According to available clinical records, the mean interval from the first documented detection of HPV16 to anal and oral sample collection was 3 and 2 years, respectively.

The overall methodological workflow from sample collection to phylogenetic analysis is shown in Figure 1.

### 2.2. DNA Extraction and HPV Typing

All anal swabs and oral rinse samples were pretreated as previously described [27,28]. DNA extraction was performed using the QIASYMPHONY automated instrument (QIAGEN, Hilden, Germany), according to the manufacturer’s instruction. Detection and typing of HPV DNA were carried out using the Allplex 28 HPV SEEGENE (SEEGENE, Seoul, Republic of Korea) real-time assay [29].

### 2.3. Characterization of HPV16 E6 and E7 Variants

For amplification of the E6 and E7 genes, the primer pairs HPV16_E6 forward (5′-CTAAGGGCGTAACCGAAATCG-3′) and HPV16_E6 reverse (5′-TGCTCATAACAGTAGAGATCAGTTG-3′) and HPV16_E7 forward (5′-CCACTGTGTCCTGAAGAA-3′) and HPV16_E7 reverse (5′-TCACCTGTATCACTGTCATT-3′) were used [30]. PCR reactions were performed in a final volume of 50 µL, containing 10 µL of sample DNA as the template, 0.5 µM of each primer, 2.5 µM of each dNTP, a buffer containing 2.5 mM MgCl_2_ (Takara, Kusatsu, Japan), and 2.5 U of HS thermostable Taq DNA polymerase (Takara, Kusatsu, Japan). The E6 and E7 regions were amplified as follows: an initial 5 min denaturation at 94 °C, then 35 cycles of 30 s at 94 °C, 45 s at 59 °C (E6)/55 °C (E7), 45 s at 72 °C, and a final extension of 7 min at 72 °C.

The resulting PCR products of approximately 600 bp for E6 and 540 bp for E7 were analyzed on 1.8% agarose gel. Positive samples were sequenced bidirectionally with the same primers used in PCR reaction using BigDye^TM^ Terminatorv3.1 Cycle Sequencing (Applied Biosystems, Waltham, MA, USA) on an automated DNA sequencer (ABI PRISM 3100 Genetic Analyzer, Applied Biosystems), following the manufacturer’s instruction.

E6 and E7 sequences were assembled using the BioEdit tool v.7.2 [31] and alignments were performed with ClustalW v.1.83. The E6/E7 concatenated sequences were submitted to GenBank (accession numbers: PX442738–PX442790).

All sequences were aligned to the prototype HPV16 genome K02718 using ClustalW in order to identify single nucleotide polymorphisms (SNPs) within E6 and E7 regions. Finally, E6 and E7 nucleotide sequences were translated into amino acid sequences to analyze the variations in the encoded proteins using the BioEdit tool. An isolate was classified as a variant if it showed at least one nucleotide substitution compared with the reference isolate [17,32].

To compare the HPV16 sequences obtained in this study with reference isolates from Italy, a search was conducted in the NCBI GenBank database using the following query: “Human papillomavirus type 16” [Organism] AND “Italy” [Country] AND (“E6” [Gene] OR “E7” [Gene]). All Italian HPV16 sequences covering the E6 and/or E7 regions were retrieved, including isolates previously reported from northern, central, and southern Italy [33,34,35]. Pairwise comparisons were then performed using the BLASTn algorithm v. 2.17.0 (https://blast.ncbi.nlm.nih.gov/Blast.cgi; accessed on 23 August 2025) to calculate the percentage of nucleotide identity between the INMI sequences and the Italian reference strains.

### 2.4. Phylogenetic Analysis

Phylogenetic analysis was conducted to determine the lineage and sublineage classification of the sequences obtained from INMI samples. All E6 sequences were aligned with reference HPV16 genomes representative of variant lineages and sublineages (A1–A4, B1–B4, C1–C4, D1–D4), retrieved from PaVE (Papillomavirus Episteme; https://pave.niaid.nih.gov/; accessed on 30 July 2025) and Galati et al. (2024) [36]. Sequence alignments were performed using ClustalW and phylogenetic trees were reconstructed in MEGA version 12 [37] using the Neighbor-Joining method and the Jukes–Cantor model, with 1000 bootstrap replicates. Lineage and sublineage (A1–A4, B1–B4, C1–C4, D1–D4) assignments were based on clustering with the corresponding reference clades, following the criteria described by Burk et al. (2013) [17] and Mirabello et al. (2018) [18]. The sequences were also categorized into sublineages according to their geographic relatedness: European (A1–A3), Asian (A4), African-1 (B1–B4), African-2 (C1–C4), North American (D1), Asian-American (D2-D3), and D4 [18]. Isolates were assigned to a specific sublineage when they clustered with the corresponding reference sequence, supported by a bootstrap value ≥70%.

To avoid redundancy, identical E6 sequences were collapsed, and one representative sequence for each group of identical isolates was included in the phylogenetic analysis, together with reference sequences of the respective sublineages. Therefore, the final dataset included 10 INMI sequences (INMI_AI12, INMI_AA4, INMI_AB5, INMI_AA7, INMI_AC10, INMI_AB6, INMI_AA2, INMI_AA1, INMI_AI14, and INMI_AD3) and 45 HPV16 reference sequences, representative of lineages A-D and sublineages A1-A4, B1-B4, C1-C4, and D1-D4 (HQ644268 A1, KU053889 A1, K02718 A1, KU053896 A2, AF536179 A2, HQ644236 A3, AF534061 A4, JQ004096 A4, KU053904 A4, HQ644234 A4, AF536180 B1, HQ644293 B1, KU053907 B1, KU053908 B1, KU053909 B2, KU053910 B2, HQ644298 B2, KU053915 B3, KU053911 B4, KU053912 B4, KU053913 B4, KU053914 B4, KU053919 C1, KU053917 C1, KU053918 C1, AF472509 C1, KU053916 C1, HQ644244 C2, KU053921 C3, KU053922 C4, KU053923 C4, KU053924 C4, KU053929 D1, KU053926 D1, HQ644257 D1, AY686579 D2, KU053940 D3, KU053942 D3, KU053941 D3, KU053944 D3, AF402678 D3, KU053943 D3, KU053933 D4, KU053934 D4, KU053931 D4).

### 2.5. Statistical Analysis

A descriptive analysis was conducted to characterize the study population. Categorical variables are expressed as frequencies (number of observations) and percentages, while continuous variables are summarized as median values with interquartile ranges (IQR). The association between E6/E7 variants and CD4+ T cell counts was assessed using the Mann–Whitney test, both in the overall cohort and within individual cytology grades (NILM, LSIL, HSIL). The Fisher–Freeman–Halton exact test was used to evaluate the relationship between E6/E7 variant distribution and cytology grade. A *p*-value less than 0.05 was considered statistically significant. All statistical analyses were performed using GraphPad Prism 10 software (GraphPad software Inc., La Jolla, CA, USA) and RStudio version 2024.09.0 software (RStudio, Boston, MA, USA).

## 3. Results

### 3.1. Study Population

A total of 53 HIV-positive MSM were included in this study, comprising 41 with HPV16 detected in anal samples and 12 in oral samples. Among them, 47 (88.7%) showed multiple HPV genotype infections: 92.7% of anal and 75.0% of oral samples (Table 1 and Table S1). In anal samples, the most prevalent genotype other than HPV16 was HPV53 (n = 16, 39.0%), followed by HPV54 and HPV68 (both detected in 13 cases, 31.7%), and HPV61 (n = 12, 29.3%). In contrast, in oral samples, the most frequent genotype was HPV82 (n = 3, 25.0%), followed by HPV70, HPV56, and HPV43 (each one found in 2 samples, 16.7%). The majority of patients (90.6%) were Italian, and the median age at sample collection was 48 (range: 29–72) years. Overall, regarding HIV virological status, HIV RNA was undetectable in the majority of individuals (56.6%), whereas 41.5% had detectable viremia below 30 copies/mL, and only one patient (1.9%) presented HIV RNA levels above 30 copies/mL (40 copies/mL). In addition, the cohort showed a median CD4+ T cell count of 681.5 cells/µL (IQR: 510.3–834), with most subjects (77.4%) exhibiting CD4+ T cell levels ≥ 500 cells/µL. No significant association was found between the distribution of E6/E7 variants and cytology grade (*p* > 0.999). CD4+ T cell counts were comparable between individuals carrying E6/E7 variants and those without variants (median 683 vs. 606 cells/µL, *p* = 0.82). When stratified by cytology grade, no significant differences were observed (NILM: *p* = 0.82; LSIL: *p* = 0.73; HSIL: *p* = 0.84). Demographic and pathological characteristics according to sample type and cytological grade are summarized in Table 1.

### 3.2. Phylogenetic Analysis

A total of 51 E6 sequences were analyzed to construct the phylogenetic tree. Among them, 26 (51.0%) carried an identical single mutation, 16 (31.4%) were identical to the reference sequence (K02718), and 3 (5.9%) shared two identical SNP patterns. The remaining 6 sequences (11.8%) showed unique SNP profiles (Supplementary Table S2).

**Figure 2:**
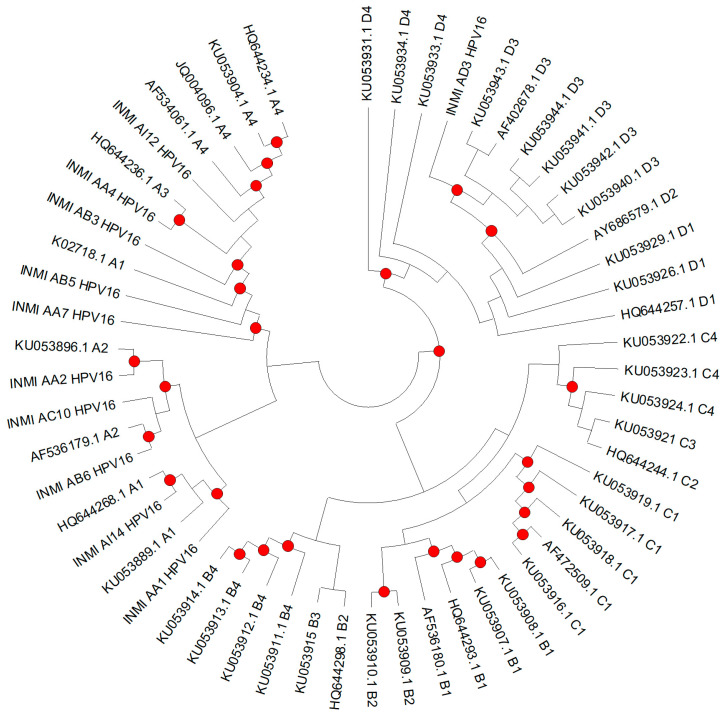
Phylogenetic analysis was performed on non-redundant datasets, with one representative sequence selected for each group of identical isolates (INMI_AI12, INMI_AA4, INMI_AB5, INMI_AA7, INMI_AC10, INMI_AB6, INMI_AA2, INMI_AA1, INMI_AI14, and INMI_AD3). Forty-five reference sequences for the different HPV16 sublineages (A1–A4, B1–B4, C1–C4, and D1–D4) were included for lineage assignment. The tree was constructed using the Neighbor-Joining method with the Jukes–Cantor model (1000 bootstrap replicates). Bootstrap values ≥70 are indicated by dots.

Since several E6 sequences were identical, one representative per group was used for phylogenetic tree construction (INMI_AI12, INMI_AA4, INMI_AB5, INMI_AA7, INMI_AC10, INMI_AB6, INMI_AA2, INMI_AA1, INMI_AI14, and INMI_AD3), along with reference sequences of the corresponding sublineages (Figure 2).

According to reference sequences, branches in the phylogenetic tree can be divided in four lineages (A–D) and further categorized according to their geographic relatedness (European, Asian, African-1, African-2, North American, and Asian-American). Overall, the phylogenetic analysis of the 51 E6 sequences showed that 96.1% clustered within the European (E) lineage (sublineages A1–3), specifically 84.3% (n = 43) in A1, 9.8% (n = 5) in A2, and 2% (n = 1) in A3. Only one patient belonged to Asian (sublineage A4) and another to Asian-American1 (sublineage D3). As shown in Table 2, most of the sequences belonged to sublineage A1 across both anal and oral samples, representing 66.7% of HSIL, 89.5% of LSIL, 80.0% of NILM anal samples, and 83.3% of oral NILM samples. Regarding anal samples, sublineage A2 was less frequent, being detected in 8.3%, 10.5%, and 10% of HSIL, LSIL, and NILM, respectively, and in 8.3% of NILM oral samples. A3 was rare (detected in one HSIL case), while A4 and D3 were each identified in a single anal NILM and anal HSIL sample, respectively. One anal HSIL and one oral NILM sample could not be assigned to a specific sublineage. No isolates corresponding to the other sublineages under investigation were identified.

### 3.3. E6 and E7 Regions

Of the 53 HPV16-positive patients, 51 HPV16 E6 and 52 HPV16 E7 sequences were successfully sequenced. Overall, pairwise similarity analysis revealed that both E6 and E7 regions were highly conserved across the HPV16 sequences analyzed. The E6 region showed a mean similarity of 99.79% (range: 98.46–100%), while E7 displayed an even higher mean similarity of 99.90% (range: 98.31–100%).

All sequences were compared with the HPV16 reference sequence K02718 to identify the presence of single nucleotide polymorphisms (SNPs) in the E6 and E7 regions. In total, 18 single-point mutations were identified: 11 in E6 and 7 in E7. Specifically, in the E6 region, 4 were synonymous and 7 non-synonymous, whereas in E7, 6 were synonymous and 1 was non-synonymous (Table 3 and Table 4). Notably, 31.4% (16/51) of E6 sequences and 90.4% (47/52) of E7 sequences showed no variation compared with the reference.

Among E6 variants, the most prevalent was T350G, resulting in the L83V amino acid change, which was detected in 62.7% (32/51) of patients and belonged to sublineages A1, A2, and D3. Interestingly, this mutation was observed across all cytological categories and in both anatomical sites, suggesting that L83V represents a common variant in this cohort, independently of lesion grade. Specifically, it was found in 63.6% of HSIL, 63.2% of LSIL and 40.0% of NILM anal samples, and 81.8% of oral NILM samples (Table 3). All other single-point mutations identified in the E6 region were sporadic, detected as single cases, and without apparent association with cytological grade or anatomical site. Five patients with the T350G also showed one additional SNP: T109C in three cases (one anal HSIL, one anal NILM, and one oral NILM sample), A131G (R10G) in one anal LSIL, and G176A (D25N) in one anal NILM. Three E6 sequences carried only a single SNP: A276G (N58S) in one anal HSIL sample (A3 sublineage), A131G (R10G) in one anal LSIL sample (A2 sublineage), and T178G (D25E) in one anal NILM sample related to the A4 sublineage. In contrast, one E6 sequence from an anal HSIL sample (D3 sublineage) harbored six single-point mutations, G145T (Q14H), T286A, A289G, C335T (H78Y), T350G (L83V), and A532G, all related to the D lineage.

Regarding the E7 region, five samples harbored single-point mutations. Among anal HSIL cases, two patients showed a single synonymous substitution, T795C and G888A, respectively. One anal HSIL sample belonging to the D3 sublineage carried three synonymous substitutions (T732C, T789C, and T795G), consistent with variants previously reported in the D lineage. One anal NILM sample presented the A647G (N29S), which is characteristic of the A4 sublineage according to the literature. Finally, one anal LSIL sample belonging to the A1 sublineage carried the T822G substitution (Table 4).

BLAST v.2.17 analysis revealed a 100% identity between several INMI sequences and Italian isolates belonging to A1 and A2 sublineages. Specifically, the INMI_AA7 sequence (sublineage A1) matched perfectly (100% identity) with MH937390.1 (Calabria, Italy, cervical sample) [34] and EF422095.1 (Lazio, Italy, cervical sample) [33]. INMI_AA1 and INMI_AA2 also showed complete identity with MH937402.1, MH937395.1 (Calabria, Italy, cervical samples) [34], and EF422093.1 (Lazio, Italy, cervical sample) [33] isolated from cervical swabs, confirming that these lineages are widespread in Italian isolates. The INMI_AA3 sequence, classified as sublineage A1, showed 99.88% identity with MH937402.1 (Calabria, Italy, cervical sample) [34], differing only by a synonymous substitution in E7 (T795C). Similarly, INMI_AA4 exhibited 99.76% identity with MH937393.1 (Calabria, Italy, cervical sample) [34], due to the presence of the G666A SNP in E7. Additionally, INMI_AC3 had 99.87% identity with MH937397.1 (Calabria, Italy, cervical sample) [34], carrying the synonymous E7 T822G variant, while INMI_AC10 shared 99.88% identity with MH937404.1 (Calabria, Italy, cervical sample) [34], presenting the E6 A131G (R10G) and T350G (L83V) amino acid changes, both previously reported in Italian A2 isolates. Finally, regarding the D3 sublineage, INMI_AD3 showed 100% identity with MH937377.1 (Lombardia, Italy, cervical sample) [35] when considering the E6 gene alone, and 99.03% identity with MH937377.1 (Calabria, Italy, cervical sample) [34] when both E6 and E7 regions were analyzed. Overall, all INMI HPV16 isolates showed a high degree of similarity (99–100% identity) with previously reported Italian variants.

## 4. Discussion

While the prevalence of HPV16 variants in cervical cancer samples from different geographical areas is well documented [38,39], less information is available on HPV variants in anal and oral samples and their contribution to HPV infection persistence. This gap in knowledge is particularly relevant, as HPV16 is the predominant high-risk genotype driving both anal and oropharyngeal carcinogenesis. To address these aspects, 51 E6 and 52 E7 HPV16 sequences from anal and oral samples of HIV-positive MSM with persistent HPV infection were analyzed to assess the HPV16 lineage distribution, oncoprotein polymorphisms, and their potential association with lesion grade and anatomical site. Lineage A was the most frequent in both anal (95.1%) and oral (91.7%) samples. The A1 sublineage was predominant (84.3%), followed by the A2 sublineage (9.8%). Only one anal specimen harbored the A4 variant (NILM), and one specimen carried the D3 variant (HSIL). No B or C lineages were observed, and no lineage coinfections were detected in either anal or oral samples. These findings are consistent with those reported in Italian cervical samples by Galati et al., where 89.7% of HPV16 strains belonged to the A1/A2 HPV16 sublineages [34], and with previous studies [40,41]. Notably, the D3 strain was associated with HSIL. The D3 sublineage has also been reported in Italian women with CIN 2 lesions and cancer and in men with penile carcinoma [25,42,43], suggesting a higher oncogenic potential for this sublineage. The L83V mutation was observed in 32/51 E6 sequences and was present in both anal (n = 23, 57.5%) and oral (n = 9, 81.8%) samples. In anal samples, its frequency was comparable between HSIL (63.6%) and LSIL (63.2%), whereas it was lower in samples with normal cytology (40%). This mutation is typical of lineage D, which appears to have greater oncogenic potential than A1 or A2 sublineages. In vitro studies have shown that keratinocytes are transformed more efficiently by the E6 oncoprotein of lineage D compared to those of lineage A [24]. However, the D lineage variants contain additional mutations besides L83V, such as Q14H and H78Y, which appear to confer greater capacity for cellular transformation and immortalization compared to the prototype E6 oncoprotein. This enhanced oncogenic potential could be linked to epistatic effects of other mutations present in different regions of the genome [19,44]. No specific E6 variant was observed in the oral cavity compared with the anal site, suggesting that there is currently no evidence of oral tropism associated with particular variants. The R10G, D25N, and N58S mutations were found only in lineage A variants. The A variants detected in the oral rinse did not present any non-synonymous mutations other than L83V (Table 3). Interestingly, three sequences harbored the synonymous T109C mutation, typical of the C lineage [43]. The prevalence of the T350G (L83V) polymorphism was similar to that reported by Galati et al. (74%) and by Tsakogiannis et al. in cervical samples [34,45]. This high prevalence may reflect regional genetic patterns or, since it was observed in samples with persistent HPV infection, may contribute to viral persistence in different anatomical sites. Notably, none of the non-synonymous mutations or amino acid variations previously associated with destabilization of the E6–EAP–p53 complex (K41E, I59V, and Q98R) or with E6 structural instability (R17T, R17I, K41E, and I59V) [46] were found in our cohort. Three mutations within immunogenic region 1, Q14H, D25N, and D25E, were found: the first in an HSIL sample, and the latter two in anal samples with normal cytology (NILM). Overall, 31.4% of the E6 sequences were identical to the HPV16 reference strain (K02718). These sequences were distributed across different cytological categories, including HSIL (n = 3), LSIL (n = 6), and NILM (n = 5) in anal samples, as well as NILM (n = 2) in oral rinse samples. As discussed, 51.0% of the analyzed samples carried the L83V (T350G) substitution, observed in both anal (HSIL = 7, LSIL = 12, and NILM = 4) and oral (NILM = 9) samples. Three additional sequences (5.9%) shared one identical single-point mutation (T109C) detected in one HSIL, one NILM anal, and one NILM oral sample. Finally, six sequences (11.8%) exhibited unique mutational profiles, suggesting that substitutions such as Q14H, R10G, D25N, D25E, N58S, and H78Y should be considered sporadic and not associated with a particular lesion grade or anatomical site. The E7 coding region showed lower variability than the E6, consistent with previous reports [20]. Six synonymous mutations were observed in three different samples: 5 in a D3-HSIL sample and 1 T822G in an A1 LSIL anal sample. Additionally, one non-synonymous N29S mutation was detected in an NILM anal sample. No polymorphisms were detected in oral samples. Information on the genetic variability of E7 is more available for cervical samples. Mirabello et al. [20] demonstrated, through NGS analysis, that women with infection without lesions had an E7 protein with greater variability than those with cervical cancer or high-grade lesions. E7 appears to be an important factor in the transformation process during the precancerous phase, when it is overexpressed [47]; therefore, its high conservation may be necessary to preserve its transforming properties. Unlike the previous observation by Zhao et al., who reported a higher frequency of N29S in the HSIL group, we identified only one N29S variant in an LSIL sample, while N29H was not observed in our samples. These apparently contradictory results could be due to the small sample size and the sublineages considered: A1-A2 in our samples, and A4 in those analyzed by Zhao et al. [48]. Moreover, statistical analysis demonstrated that the variability of E6 and E7 was not associated with CD4+ T cell count or HIV RNA viral load. This finding suggests that, within our cohort of HIV-positive MSM, viral genetic variability appears to be independent of immune control parameters.

Our study has several limitations. The reference sequence K02718 was used in this study because it represents the internationally accepted consensus for HPV16 and allows our data to be compared with those reported in other studies adopting the same reference. On the other hand, it would be desirable to include sequences derived from oral or anal sites of HIV-negative individuals to assess whether the observed variability may also be influenced by HIV serostatus. Furthermore, the limited sample size does not allow firm conclusions on the impact of E6 and E7 mutations on infection persistence. Nevertheless, our data confirm that E6 shows greater variability than E7 in both anal and oropharyngeal samples and that these mutations are not associated with high-grade lesions or site-specific tropism. Unfortunately, transient HPV16 infections were not included, because their number was negligible. This finding is consistent with the well-documented tendency of HPV16 to persist in most cases, as shown in the HIM cohort, where 80% of HPV16 infections detected at baseline persisted for 24 months [49]. Consequently, transient HPV16 infections were not observed in our cohort and were therefore not included in this study. In conclusion, this study is the first to analyze the variability of HPV16 E6 and E7 oncoproteins in the oral and anal districts of HIV-positive MSM. Our findings highlight the absence of site-specific variants, and the lack of association between genetic variability and immune parameters, suggesting no evidence of variant-specific tropism in this population.

## Figures and Tables

**Table 1:** Characteristics of the study population; N = 53.

	Total (N = 53)	Anal Samples (n = 41)			Oral Samples (n = 12)
		HSIL (n = 12)	LSIL (n = 19)	NILM (n = 10)	NILM (n = 12)
**Age,** **median years (range)**	48 (29–72)	45 (39–69)	51 (34–72)	46 (29–60)	47 (35–64)
**Ethnicity, n (%)** **Italian** **Not Italian**	48 (90.6) 5 (9.4)	10 (83.3) 2 (16.7)	18 (94.7) 1 (5.3)	9 (90.0) 1 (10.0)	11 (91.7) 1 (8.3)
**Multiple alpha-HPV infection, n (%)**	47 (88.7)	12 (100.0)	18 (94.7)	8 (80.0)	9 (75.0)
**HIV RNA, n (%)** **Not detected** **Detectable:** **<30 copies/mL** **>30 copies/mL**	30 (56.6) 22 (41.5) 1 (1.9)	4 (33.3) 8 (66.7) 0	16 (84.2) 2 (10.5) 1 (5.3)	4 (40.0) 6 (60.0) 0	6 (50.0) 6 (50.0) 0
**CD4+ T cell count,** **median cells/μL (IQR)** **≥500/μL, n (%)** **201–499/μL, n (%)** **≤200/μL, n (%)**	681.5 (510.3–834) 41 (77.4) 11 (20.8) 1 (1.8)	484 (374–797) 6 (50.0) 5 (41.7) 1 (8.3)	571 (468–933) 14 (73.7) 5 (26.3) 0	671 (520–757) 9 (90.0) 1 (10.0) 0	722 (687–834) 12 (100.0) 0 0

Abbreviations: IQR, interquartile range; HSIL, high-grade intraepithelial lesion; LSIL, low-grade intraepithelial lesion; NILM, negative for intraepithelial lesion and malignancy. This information was obtained from clinical records.

**Table 2:** HPV16 sublineage distribution according to cytology results.

	Total (N = 53)	Anal Samples (n = 41)			Oral Samples (n = 12)
**HPV16** **Sublineages**		HSIL (n = 12)	LSIL (n = 19)	NILM (n = 10)	NILM (n = 12)
**A1, n (%)** **A2, n (%)** **A3, n (%)** **A4, n (%)** **D3, n (%)** **Not available**	43 (81.1) 5 (9.4) 1 (1.9) 1 (1.9) 1 (1.9) 2 (3.8)	8 (66.7) 1 (8.3) 1 (8.3) - 1 (8.3) 1 (8.3)	17 (89.5) 2 (10.5) - - - -	8 (80.0) 1 (10.0) - 1 (10.0) - -	10 (83.3) 1 (8.3) - - - 1 (8.3)

Abbreviations: HSIL, high-grade intraepithelial lesion; LSIL, low-grade intraepithelial lesion; NILM, negative for intraepithelial lesion and malignancy.

**Table 3:** Distribution of detected single-point mutations in HPV16 E6 gene according to lesion grade in INMI samples.

	E6										
**K02718**	T	A	G	G	T	A	T	A	C	T	A
**Position**	109	131	145	176	178	276	286	289	335	350	532
	C	G	T	A	G	G	A	G	T	G	G
**AA change**	-	R10G	Q14H	D25N	D25E	N58S	-	-	H78Y	L83V	-
**HSIL** **Anal samples** **(n = 11)**	1 (9.1)		1 (9.1)			1 (9.1)	1 (9.1)	1 (9.1)	1 (9.1)	7 (63.6)	1 (9.1)
**LSIL** **Anal samples** **(n = 19)**		2 (10.5)								12 (63.2)	
**NILM** **Anal samples** **(n = 10)**	1 (10.0)			1 (10.0)	1 (10.0)					4 (40.0)	
**NILM** **Oral samples** **(n = 11)**	1 (9.1)									9 (81.8)	

The nucleotide position of each single-point mutation was indicated according to the numbering of the HPV16 K02718 sequence. Data are expressed as number of samples and (%) harboring each specific E6 point mutation within the indicated lesion grade; individual samples may carry more than one mutation. AA change, amino acid changes; HSIL, high-grade intraepithelial lesion; LSIL, low-grade intraepithelial lesion; NILM, negative for intraepithelial lesion and malignancy.

**Table 4:** Distribution of detected single-point mutations in HPV16 E7 gene according to lesion grade in INMI samples.

	E7						
**K02718**	A	G	T	T	T	T	T
**Position**	647	666	732	789	795	795	822
	G	A	C	C	C	G	G
**AA change**	N29S	-	-	-	-	-	-
**HSIL** **Anal samples** **(n = 12)**		1 (8.3)	1 (8.3)	1 (8.3)	1 (8.3)	1 (8.3)	
**LSIL** **Anal samples** **(n = 19)**							1 (5.3)
**NILM** **Anal samples** **(n = 10)**	1 (10.0)						
**NILM** **Oral samples** **(n = 12)**							

The nucleotide position of each single-point mutation was indicated according to the numbering of the HPV16 K02718 sequence. Data are expressed as number of samples and (%) harboring each specific E7 point mutation within the indicated lesion grade; individual samples may carry more than one mutation. AA change, amino acid changes; HSIL, high-grade intraepithelial lesion; LSIL, low-grade intraepithelial lesion; NILM, negative for intraepithelial lesion and malignancy.

## Data Availability

Sequences were submitted to GenBank (accession numbers: PX442738–PX442790). The data presented in this study are available on request from the corresponding author.

## Supplementary Materials

The following supporting information can be downloaded at https://www.mdpi.com/article/10.3390/pathogens14121210/s1, Table S1: Summary of HPV16-positive patients (N = 53) according to cytological grade and alpha-HPV co-infection pattern; Table S2: Grouping of identical HPV16 E6 sequences based on mutational profiles identified in anal and oral samples.
