# Supplementary material for: HPV16 E6 and E7 Genetic Variability in Oral and Anal Samples from HIV-Positive MSM
Source: Pathogens. 2025 Nov 28;14(12):1210. doi: 10.3390/pathogens14121210 (PMC12736272; doi:10.3390/pathogens14121210)
Supplement: Supplementary file 1 [file pathogens-14-01210-s001.zip › pathogens-4010741-supplementary.pdf]

Supplementary Table S1. Summary of HPV16-positive patients (N=53) according to cytological grade and alpha-HPV co-infection pattern.

Patient ID	Sample	Cytological grade	Multiple alpha-HPV infection
INMI_AA1	Anal	HSIL	16, 42, 53
INMI_AA2	Anal	HSIL	66,58,16,52,35,68,31,69,616,11
INMI_AA3	Anal	HSIL	51,16,39,43
INMI_AA4	Anal	HSIL	16,45,52,54,6,61,70
INMI_AA5	Anal	HSIL	16,31,35,51,52,61,68,69,70,73,82
INMI_AA7	Anal	HSIL	16,42,43,44,51,44,51,56,58,6,68,69,73
INMI_AB1	Anal	HSIL	16,31,51,52,53,58,6,61,66
INMI_AB2	Anal	HSIL	16,33,53,54,66,68,82
INMI_AB3	Anal	HSIL	66,58,16,39,18,68,26,69,42,53,70,61,6
INMI_AD1	Anal	HSIL	11,16,45,53,6,66,68,70
INMI_AD2	Anal	HSIL	16,33,45,6
INMI_AD3	Anal	HSIL	16,52,58,66,70
INMI_AA9	Anal	LSIL	16,35,56,31
INMI_AA10	Anal	LSIL	59,16,31,69,73,42,53,43,11
INMI_AB4	Anal	LSIL	11,16
INMI_AB5	Anal	LSIL	16,44,45,52,54,59
INMI_AB6	Anal	LSIL	16,35,54,68,82
INMI_AB7	Anal	LSIL	16,35,82,53,54,70,6
INMI_AB8	Anal	LSIL	16,33,42,54,61,69
INMI_AC1	Anal	LSIL	16,58,33,39,35
INMI_AC2	Anal	LSIL	59,16,68,82,61
INMI_AC3	Anal	LSIL	16,39,35,42,53,54,61,6
INMI_AC4	Anal	LSIL	16,18,56,53,61,44
INMI_AC5	Anal	LSIL	16,56,68,54
INMI_AC7	Anal	LSIL	58,16,5,42,70,44
INMI_AC8	Anal	LSIL	16,56,68,54
INMI_AC10	Anal	LSIL	16,18,73,42,6
INMI_AC11	Anal	LSIL	16
INMI_AD5	Anal	LSIL	16,44,70
INMI_AD9	Anal	LSIL	51,16,69,73,42,82,53,70
INMI_AD10	Anal	LSIL	45,16,68,31,42,44
INMI_AI1	Anal	NILM	16,39,42,43,44,53,54,58,73
INMI_AI2	Anal	NILM	16
INMI_AI5	Anal	NILM	66,51,16,39,52,56,31,26,42,53,61,44,40
INMI_AI6	Anal	NILM	59,16,18,26,82,53,70,40
INMI_AI8	Anal	NILM	16
INMI_AI9	Anal	NILM	16,31,53,44
INMI_AI10	Anal	NILM	51,16,39,68,53,54,61
INMI_AI11	Anal	NILM	58,16,73,54,70
INMI_AI12	Anal	NILM	58,16,52,11
INMI_AI14	Anal	NILM	16,68,53,54,61,44

INMI_OP1	Oral	NILM	16,82
INMI_OP2	Oral	NILM	16,66
INMI_OP3	Oral	NILM	16,56,70
INMI_OP4	Oral	NILM	16,43
INMI_OP5	Oral	NILM	16,82
INMI_OP7	Oral	NILM	16,33
INMI_OP8	Oral	NILM	16
INMI_OP9	Oral	NILM	16
INMI_OP10	Oral	NILM	16
INMI_OG1	Oral	NILM	16,56,70
INMI_OG3	Oral	NILM	11,16,43
INMI_OG5	Oral	NILM	16,82

Abbreviations: HSIL, high grade intraepithelial lesion; LSIL, low grade intraepithelial lesion; NILM, negative for intraepithelial lesion and malignancy.

Supplementary Table S2. Grouping of identical HPV16 E6 sequences based on mutational profiles identified in anal and oral samples.

<table>
<tr><th>Group ID</th><th>Sample IDs (identical sequences)</th><th>Total</th><th>Anal</th><th>Oral</th><th>Anal HSIL</th><th>Anal LSIL</th><th>Anal NILM</th><th>Oral NILM</th></tr>
<tr><td>1[a]</td><td>INMI_AA1</td><td rowspan="26">26</td><td rowspan="26">18</td><td rowspan="26">8</td><td rowspan="26">5</td><td rowspan="26">11</td><td rowspan="26">2</td><td rowspan="26">8</td></tr>
<tr><td>1</td><td>INMI_AA3</td></tr>
<tr><td>1</td><td>INMI_AA5</td></tr>
<tr><td>1</td><td>INMI_AB1</td></tr>
<tr><td>1</td><td>INMI_AD1</td></tr>
<tr><td>1</td><td>INMI_AA9</td></tr>
<tr><td>1</td><td>INMI_AA10</td></tr>
<tr><td>1</td><td>INMI_AB4</td></tr>
<tr><td>1</td><td>INMI_AB7</td></tr>
<tr><td>1</td><td>INMI_AB8</td></tr>
<tr><td>1</td><td>INMI_AC1</td></tr>
<tr><td>1</td><td>INMI_AC3</td></tr>
<tr><td>1</td><td>INMI_AC4</td></tr>
<tr><td>1</td><td>INMI_AC8</td></tr>
<tr><td>1</td><td>INMI_AD5</td></tr>
<tr><td>1</td><td>INMI_AD9</td></tr>
<tr><td>1</td><td>INMI_AI2</td></tr>
<tr><td>1</td><td>INMI_AI6</td></tr>
<tr><td>1</td><td>INMI_OP1</td></tr>
<tr><td>1</td><td>INMI_OP2</td></tr>
<tr><td>1</td><td>INMI_OP3</td></tr>
<tr><td>1</td><td>INMI_OP4</td></tr>
<tr><td>1</td><td>INMI_OP5</td></tr>
<tr><td>1</td><td>INMI_OG1</td></tr>
<tr><td>1</td><td>INMI_OG3</td></tr>
<tr><td>1</td><td>INMI_OG5</td></tr>
</table>

2[b]	INMI_AA7	16	14	2	3	6	5	2
2	INMI_AB3							
2	INMI_AD2							
2	INMI_AB5							
2	INMI_AC2							
2	INMI_AC5							
2	INMI_AC7							
2	INMI_AC11							
2	INMI_AD10							
2	INMI_AI1							
2	INMI_AI5							
2	INMI_AI8							
2	INMI_AI9							
2	INMI_AI11							
2	INMI_OP7							
2	INMI_OP8							
3[c]	INMI_AA2	3	2	1	1	0	1	1
3	INMI_AI10							
3	INMI_OP10							
4[d]	INMI_AA4	6	6	0	2	2	2	0
4	INMI_AD3							
4	INMI_AB6							
4	INMI_AC10							
4	INMI_AI12							
4	INMI_AI14							

1[a]: sequences carrying the single L83V (T350G) mutation with no additional variations.

2[b]: sequences completely identical to the HPV16 reference genome (K02718).

3[c]: sequences sharing the same SNP pattern.

4[d]: unique sequences, each displaying a distinct SNP profile.
